# Continuous glucose monitoring assessment of metabolic control in east African children and young adults with type 1 diabetes: A pilot and feasibility study

**DOI:** 10.1002/edm2.135

**Published:** 2020-06-08

**Authors:** Lauren McClure Yauch, Eric Velazquez, Thereza Piloya‐Were, Lucy Wainaina Mungai, Anjumanara Omar, Antoinette Moran

**Affiliations:** ^1^ Department of Pediatrics University of Minnesota Minneapolis MN USA; ^2^ Department of Pediatrics Makerere University College of Health Sciences Kampala Uganda; ^3^ Department of Pediatrics University of Nairobi Nairobi Kenya

**Keywords:** continuous glucose monitoring, healthcare disparity, hypoglycaemia, low resource nations

## Abstract

**Background:**

For individuals with type 1 diabetes (T1D) in East Africa and other low‐income regions, the last decade has seen substantial gains in access to insulin and trained healthcare providers, yet metabolic control remains poor.

**Methods:**

The objective was to determine the feasibility of continuous glucose monitoring (CGM) and to gather baseline metabolic data for future power analysis in Ugandan and Kenyan youth with T1D using a Freestyle Libre Pro blinded CGM.

**Results:**

Of 78 participants recruited, four sensors fell off and six patients did not return, leaving 68 evaluable subjects. Average age was 16 ± 5 (range 4‐26) years, 43% female. Average diabetes duration was 7 ± 5 years, insulin dose 0.9 ± 0.3 U/kg/d, and number of fingerstick glucose levels per day 2.1 ± 1.1. All were on human insulin. Point‐of‐care HbA1c was 10.9 ± 2.7% (96 ± 30 mmol/mol). Mean number of sensor days was 13 ± 3; >90% wore the sensor for ≥10 days. Mean glucose was 231 ± 86 mg/dL (12.8 ± 4.8 mmol/L). Only 30 ± 19% of time was spent in the target range (70‐180 mg/dL; 3.9‐10 mmol/L), and 7 ± 8% of time was spent in hypoglycaemia (glucose <55 mg/dL, 3.0 mmol/L). Hypoglycaemia occurred in 81% of participants, averaging five events/wk with an average duration of 140 ± 79 minutes/event.

**Conclusions:**

Despite significant diabetes care improvements, East African youth with T1D have poor metabolic control with chronic hyper‐ and hypoglycaemia, placing them at high risk for serious acute and chronic complications. This study demonstrates the feasibility of CGM use in this population and provides baseline metabolic data that will be used to inform a future intervention study.

## Graphical abstract

Despite significant improvements in diabetes care, continuous glucose monitoring demonstrates that youth with T1D in East Africa have poor metabolic control with chronic hyper‐ and hypoglycaemia, placing them at high risk for serious acute and chronic complications. Current methods of care are inadequate in this population.

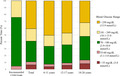

## 1 INTRODUCTION

In 2017, the International Diabetes Federation reported that 50 600 children aged 0‐19 were known to be living with type 1 diabetes (T1D) in Africa, with about 18 300 new cases diagnosed each year.^1^ Major obstacles to providing T1D care have included lack of trained healthcare professionals, poor availability of insulin and testing supplies, and limited patient education materials. These same issues are common in much of the resource‐poor world. While there are still problems in East Africa, all of these issues have substantially improved over the last decade.

The Pediatric Endocrinology Training Centre for Africa (PETCA) fellowship programme was established in Kenya in 2008 by a partnership between local hospitals, the European Society for Pediatric Endocrinology, the World Diabetes Foundation (WDF) and the International Society for Pediatric and Adolescent Diabetes (ISPAD).^2^ Prior to 2008, there were only a few trained paediatric endocrinologists in all of Africa; as of July 2019, 87 graduates of this programme were practicing in 14 African countries including three in Uganda and 12 in Kenya.

Programmes such as Changing Diabetes in Children (CDiC, a collaboration between Novo Nordisk, the World Diabetes Foundation, Roche Pharmaceuticals and local health ministries), Life for a Child and local African partner organizations now ensure that reliable and sufficient quantities of human insulin are usually available to children in Uganda and Kenya, and, to a lesser extent, home glucose metres, glucose test strips and haemoglobin A1c (HbA1c) testing supplies are provided. Nurses, dietitians and pharmacists have been trained, dedicated diabetes clinics have been established, patient education materials have been made freely available in multiple local languages, a guidebook for healthcare professionals on T1D in children is available and widely used, and an electronic patient registry has been developed and distributed.

Despite these tremendous improvements in diabetes care delivery, the available HbA1c data show that the expected positive impact on glycaemic control has not occurred, and HbA1c levels remain unacceptably high in sub‐Saharan Africa. This suggests that a fresh intervention approach is needed.^3^, ^4^, ^5^, ^6^ While HbA1c is a well‐accepted measure of overall diabetes control, it does not provide other important measures such as the per cent time spent in hypoglycaemic or hyperglycaemic glucose ranges, glycaemic variability or daily glycaemia patterns. The current study was performed to explore these measures of glucose metabolic control, using blinded continuous glucose monitoring (CGM) in African patients with T1D attending the paediatric diabetes clinics at national referral hospitals in Uganda and Kenya. These novel data will inform the power analysis for a planned intervention study. An additional goal was to determine the feasibility of wearing CGM in this population. The equatorial climate is hot and humid, raising concerns about sensor skin adhesion or skin irritation. There were also concerns about whether patients would accept wearing this visible evidence of diabetes in a region where the disease is a social stigma.

## 2 METHODS

### 2.1 Study design

The primary purpose of this cross‐sectional observational study was to characterize glucose metabolic control in children and youth with T1D in East Africa using the blinded Freestyle Libre Pro CGM system (Abbott Diabetes Care), in order to obtain data for power analysis for a future intervention study. We hypothesized that time‐in‐range would be lower than that recommended, and time‐in‐hypoglycaemia higher. A second objective was to determine the feasibility of CGM in this population. The Uganda and Kenya sites were chosen because of their long‐standing working relationships with the paediatric diabetes team at the University of Minnesota.

### 2.2 The Freestyle Libre pro flash CGM system

The Freestyle Libre is an intermittently scanned (or ‘flash’) CGM system that is factory calibrated and can be worn for up to 14 days without the need for additional calibrations.^7^ The Freestyle Libre Pro is a blinded CGM. Glucose readings are stored on the sensor with no real‐time data available to the patient, who does not need to do anything but wear the device. Data are automatically stored and then uploaded once the sensor is removed, and a retrospective report is generated for the medical professional. It has been suggested that this system is less accurate in the low glucose range than in normal or high glucose ranges, with one group reporting a hypoglycaemia MARD (mean of the absolute value of the difference between the CGM value and the reference value, divided by the reference value) of up to 24%.^8^, ^9^ This means a CGM value of 55 mg/dL (3.0 mmol/L) could be on average 24% lower or higher than the laboratory assessment, or between 42 and 68 mg/dL (2.3‐3.8 mmol/L), with just as much chance that the number could be off in one direction as the other. However, that was a small study with only 56 comparisons of CMG vs measured glucose levels. Other studies have suggested a high degree of accuracy with the FreeStyle Libre CGM, including at low glucose ranges.^10^, ^11^, ^12^

### 2.3 Participants

Participants were recruited from the paediatric endocrinology clinics at Mulago Hospital in Kampala, Uganda and Kenyatta Hospital in Nairobi, Kenya, using convenience single‐stage sampling methods. Inclusion criteria were age 2‐26 years with >6 months of duration of T1D. Patients were studied in November‐December of 2018 in Uganda, when members of the University of Minnesota (UMN) paediatric diabetes team were physically present to assist with the study. While the UMN team trained the Kenyan investigators during that same time period, due to delays in institutional review board (IRB) approval the Kenyan subjects were studied in February and March 2019, after the Minnesota team had left.

### 2.4 Procedure

At enrolment, participants completed a survey including questions about demographics, diabetes history and current diabetes management. Participants reported any history of severe hypoglycaemia events in the last year and the frequency. Severe hypoglycaemia was defined as an event where individuals were cognitively impaired to the point that they were unable to treat themselves; were unable to verbalize their needs; were incoherent, disoriented and/or combative; or experienced seizure or loss of consciousness.

After completing the survey, all participants had point‐of‐care HbA1c levels measured. The analyser at Mulago Hospital was a Hemocue HbA1c 501 System, with a reported coefficient of variation (CV) of 1.7%‐5.5%.^13^ The analyser at Kenyatta Hospital was a Siemens DCA Vantage, CV 2.5%.^14^ Analyser quality controls were run on each day of use.

The Minnesota team worked directly with the Uganda team to place the FreeStyle Libre Pro CGM sensor on each participant and trained the Kenyan team to do so themselves as their study was to take place later. The sensor was primarily placed on the back of the upper arm; two participants had sensors placed on the upper buttocks due to lack of sufficient subcutaneous arm fat. Skin Tac Adhesive Barrier Wipes (Torbot Group, Inc) were used as an adhesive, and subjects were also given Coban Self‐Adherent Wrap (3M, Minnesota) to use if needed for extra adhesion. The clinic was supplied with Tac Away Adhesive Remover (Torbot Group, Inc) for end‐of‐study sensor removal.

Participants were asked to wear the FreeStyle Libre Pro sensor for 2 weeks. CGM blood glucose levels were blinded to participants and investigators during this time. Participants were instructed to manage their diabetes normally, administering their daily insulin doses and checking their blood glucose level using home glucose metres per their usual routine, eating their typical diet and participating in their usual activities. They received a notebook and pens and were asked to record daily capillary blood glucose concentrations whenever they measured them, time and amount of insulin doses received, and to log other events such as hypoglycaemia symptoms, severe hypoglycaemia, hospitalization, or additional health visits or treatment. At the end of 2 weeks, participants returned to clinic. Researchers removed the sensor (or subjects brought it with them if it had fallen off at home) and uploaded the glucose data to LibreView, an online website that stores the sensor data and creates a report. The reports were then downloaded for data analysis by the research team, and for clinical decision‐making by the local teams.

### 2.5 Data analysis

Analysis of demographics and subject characteristics (age, gender and glycaemic metrics) was descriptive and used the data analysis program IBM SPSS 25. Summary statistics were calculated, including mean and standard deviation, and because the data were non‐normally distributed, median and interquartile range (IQR). HbA1c results that were >14% (130 mmol/mol, the upper limit of measurement for the testing devices) were recorded as 15% (140 mmol/mol) for analysis purposes. A single‐factor analysis of variance (ANOVA) was completed to evaluate for differences in mean blood glucose levels between age groups, duration of diabetes, insulin type and gender. Significance was accepted at *P* < .05. As there were no gender differences in age (*P* = .81), duration of diabetes (*P* = .75), HbA1c (*P* = .54) or glucose time‐in‐range (*P* = .47), the data were not separated by gender.

Regression analysis was performed using CGM outcomes as the dependent variable to determine whether duration of diabetes, insulin regimen, age, duration of diabetes and age at diagnosis were associated with the glycaemic data.

## 3 RESULTS

### 3.1 Baseline characteristics

#### 3.1.1 Patient demographics

In Uganda, 62 participants were enrolled. Five did not return their sensors (8%), and one fell off within 72 hours and was not included in the data analysis, leaving 56 subjects with evaluable data. In Kenya, 16 sensors were placed on eligible subjects. Three fell off within 1‐2 days due to adhesive problems and were not included in the analysis. One patient (6%) did not return the sensor, leaving 12 subjects with evaluable data.

Participant characteristic are listed in Table 1. In Uganda, the average age of participants was 16 ± 5 years. Fifty‐four per cent were female, 23% were age 4‐11 years, 32% age 11‐17, and 45% age 18‐26. This age distribution is roughly representative of the Ugandan paediatric diabetes clinic population.^3^ In Kenya, there were five children age 4‐17 and seven youth age 18‐26, with one‐third of subjects male. Patients were normally nourished in both countries.

**TABLE 1:** Baseline characteristics of study participants, mean ± SD [median; range], N = 68

	All ages	Age 4‐11	Age 12‐17	18‐26
Uganda				
Number	56	13	18	25
Age (y)	16 ± 5 [17;21]	9 ± 2 [9;7]	15 ± 1 [15;4]	20 ± 2 [20;7]
Gender	26 M/30F	8M/5F	5M/13F	13M/12F
BMI kg/m^2^	20.1 ± 3.8 [20;20]	16.2 ± 2.3 [16;8]	19.5 ± 2.9 [20;9]	22.6 ± 3.2 [21;13]
Age at diagnosis (y)	9 ± 4 [9;15]	5 ± 3 [3;8]	10 ± 5 [11;15]	10 ± 3 [10;13]
Duration diabetes (y)	7 ± 5 [8;18]	4 ± 3 [3;8]	5 ± 4 [4;12]	10 ± 4 [10;17]
Insulin dose (U/kg/d)	0.9 ± 0.3 [0.9;1.2]	0.9 ± 0.3 [0.9;1.2]	0.9 ± 0.3 [0.9;1.0]	0.9 ± 0.2 [0.8;1.0]
SMBG test number/d	2.2 ± 1.1 [2;4]	2.0 ± 0.7 [2;3]	2.4 ± 1.2 [3;4]	2.1 ± 1.1 [2;4]
Kenya				
Number	12	2	3	7
Age (y)	18 ± 6 [21;17]	10 ± 1 [10;2]	13 ± 1 [13;1]	22 ± 2 [21;6]
Gender	3M/9F	0M/2F	2M/1F	1M/6F
BMI (kg/m^2^)	21.0 ± 4.6 [20.2;17.7]	19.3 ± 1.6 [19.3;2.3]	17.1 ± 1.6 [17.3;3.1]	23.2 ± 4.8 [21.6;14.4]
Age at diagnosis (y)	11 ± 6 [11;21]	7 ± 5 [7;8]	4 ± 4 [3;7]	15 ± 5 [15;15]
Duration diabetes (y)	7 ± 4 [6;14]	3 ± 4 [3;6]	9 ± 4 [11;7]	7 ± 4 [5;11]
Insulin dose (U/kg/d)	0.7 ± 0.3 [0.7;0.8]	0.6 ± 0.1 [0.6;0.2]	1.0 ± 0.1 [1.0;0.2]	0.7 ± 0.2 [0.7;0.8]
SMBG test number/d	1.6 ± 1.0 [1;3]	1.5 ± 0.7 [2;1]	1.3 ± 1.5 [1;3]	1.7 ± 1.0 [1;2]
Combined data				
Number	68	15	21	32
Age (y)	16 ± 5 [17;22]	9 ± 2 [9;7]	15 ± 2 [14;5]	21 ± 2 [21;8]
Gender	29 M/39F	8M/7F	7M/14F	14M/18F
BMI (kg/m^2^)	20.3 ± 4.0 [20.1;21.3]	16.6 ± 2.4 [16.5;8.5]	19.2 ± 2.8 [18.6;9.4]	22.8 ± 3.5 [21.7;14.4]
Age at diagnosis (y)	9 ± 5 [10;22]	5 ± 3 [3;10]	9 ± 5 [9;15]	11 ± 4 [12;20]
Duration diabetes (y)	7 ± 5 [8;19]	4 ± 3 [3;9]	6 ± 4 [5;12]	10 ± 4 [10;17]
Insulin dose (U/kg/d)	0.9 ± 0.3 [0.8;1.3]	0.9 ± 0.3 [0.8;1.2]	0.9 ± 0.2 [0.9;1.0]	0.8 ± 0.3 [0.8;1.2]
SMBG test number/d	2.1 ± 1.1 [2;4]	1.9 ± 0.7 [2;3]	2.3 ± 1.3 [3;4]	2.0 ± 1.1 [2;4]

Abbreviations: BMI, body mass index; SMBG, self‐monitoring of blood glucose by fingerstick.

Characteristics of the 10 subjects (13%) who did not complete the study were analysed and compared to completers. No statistically significant difference was found in age, gender, time with diabetes, age at diagnosis or point‐of‐care HbA1c compared to participants who completed the study (all *P* values >.05).

#### 3.1.2 Baseline diabetes characteristics

In both countries, the average diabetes duration was about 7 years and the average insulin dose was 0.9 ± 0.3 units per kg body weight. All participants received human insulin, including NPH (isophane), regular (soluble) and Mixtard (premixed 70% NPH and 30% regular insulin). Insulin usage was similar between the two countries with the majority of patients (n = 43, 63%) on Mixtard at the time of the study, with some of these (n = 11, 16%) receiving an extra noon injection of regular insulin. The remaining 25 subjects (37%) received twice daily NPH insulin plus two (n = 12), or three (n = 3) injections of regular insulin. There was no statistically significant relation between insulin type (separate NPH and regular versus premixed insulin) and either mean glucose (*P* = .76) or HbA1c (*P* = .78) level. There was also no significant relation between the duration of diabetes and mean glucose (*P* = .34) or HbA1c level (*P* = .72). Patients tested blood glucose levels at home an average of 2.2 ± 1.1 times per day in Uganda and 1.6 ± 1.0 times per day in Kenya. Across both countries, 78% of patients reported being able to test blood glucose at least twice daily, while 13% reported no daily blood glucose testing.

### 3.2 Glucose metabolic data

Glucose metabolic data are shown in Table 2. Sensors were worn on average 13 ± 3 days (range 4‐15 days), with 90% of participants wearing it for at least 10 days. The average mean glucose level was 231 ± 86 mg/dL (12.8 ± 5.4 mmol/L) for all participants, with a trend towards lower averages in the young adult group, although this did not reach statistical significance; duration of diabetes also did not have a significant impact on the results.

**TABLE 2:** Glucose metabolic characteristics of study participants, mean ± SD [median; range], N = 68

	All Ages	Age 4‐11	Age 12‐17	18‐26
Uganda				
N	56	13	18	25
HbA1c, (%)	11.3 ± 2.7 [11.2;9.3]	11.4 ± 3.2 [11.2;9.3]	12.4 ± 3.0 [13.4;8.5]	10.4 ± 1.8 [10.0;7.1]
HbA1c (mmol/mol)	99.8 ± 29.1 [98.9;101.7]	100.9 ± 34.5 [98.9;101.7]	112.0 ± 32.7 [123.0;92.9]	90.4 ± 19.5 [85.8;77.6]
Days of sensor data	14 ± 1.9 [15;9]	13.5 ± 2.7 [15;9]	14.1 ± 2.1 [15;7]	14.1 ± 1.3 [15;5]
Mean CGM glucose (mg/dL)	239.8 ± 84.8 [225;336]	265.7 ± 103.1 [311;336]	260.6 ± 93.6 [250;298]	211.4 ± 58.6 [208;217]
CGM coefficient of variation	50 ± 22 [44;122]	50 ± 34 [39;122]	52 ± 24 [43;97]	50 ± 13 [47;59]
% time ≥250 mg/dL (13.9 mmol/L)	43 ± 27 [41;47]	52 ± 33 [64;50]	49 ± 28 [48;40]	34 ± 21 [36;28]
% time 181‐249 mg/dL (10.1‐13.8 mmol/L)	18 ± 8 [18;10]	13 ± 10 [12;10]	17 ± 7 [16;10]	21 ± 6 [22;8]
% time 70‐180 mg/dL (3.9‐10.0 mmol/L)	29 ± 18 [29;23]	27 ± 24 [16;23]	26 ± 18 [25;24]	33 ± 13 [31;17]
% time 55‐69 mg/dL (3.1‐3.8 mmol/L)	4 ± 4 [3;4]	4 ± 6 [2;4]	4 ± 3 [3;5]	4 ± 3 [4;3]
% time <55 mg/dL (3.0 mmol/L)	6 ± 7 [3;9]	4 ± 4 [4;6]	6 ± 9 [1;8]	7 ± 7 [5;9]
Kenya				
N	12	2	3	7
HbA1c, (%)	9.1 ± 1.8 [8.9;5.6]	9.2 ± 1.7 [9.2;2.4]	9.7 ± 1.0 [10.0;1.9]	8.8 ± 2.2 [8.0;5.6]
HbA1c (mmol/mol)	75.9 ± 19.9 [73.8;61.2]	77.0 ± 18.6 [77.0;26.2]	82.5 ± 10.8 [85.8;20.8]	72.7 ± 22.4 [63.9;61.2]
Days of sensor data	10.8 ± 4.2 [13.5;11.0]	7.0 ± 1.4 [7.0;2.0]	10.7 ± 5.8 [14.0;10.0]	12.0 ± 3.8 [14.0;9.0]
Mean CGM glucose (mg/dL)	188.3 ± 82.6 [208;229]	221.0 ± 33.9 [221;48]	219 ± 81.5 [218;163]	165.7 ± 93.2 [132;229]
CGM coefficient of variation	39 ± 9 [39;30]	35 ± 7 [35;10]	43 ± 15 [43;30]	39 ± 6 [38;20]
% time ≥250 mg/dL (13.9 mmol/L)	29 ± 29 [27;51]	36 ± 27 [36;19]	39 ± 34 [39;34]	23 ± 30 [0;43]
% time 181‐249 mg/dL (10.1‐13.8 mmol/L)	20 ± 13 [22;12]	35 ± 14 [35;10]	20 ± 6 [23;5]	16 ± 13 [21;19]
% time 70‐180 mg/dL (3.9‐10.0 mmol/L)	35 ± 24 [32;40]	24 ± 19 [24;14]	31 ± 33 [20;32]	40 ± 23 [44;33]
% time 55‐69 mg/dL (3.1‐3.8 mmol/L)	7 ± 9 [2;9]	2 ± 1 [2;1]	2 ± 3 [2;3]	11 ± 11 [7;18]
% time <55 mg/dL (3.0 mmol/L)	9 ± 10 [7;16]	4 ± 6 [4;4]	8 ± 8 [8;8]	11 ± 12 [6;20]
Combined data				
N	68	15	13.6	2.9
HbA1c, (%)	10.9 ± 2.7 [10.5;9.3]	11.1 ± 3.1 [11.0;9.3]	12.0 ± 2.9 [13.0;8.5]	10.1 ± 2.0 [10.0;7.3]
HbA1c (mmol/mol)	95.6 ± 29.0 [90.7;101.7]	97.7 ± 33.4 [96.7;101.7]	107.8 ± 32.1 [118.6;92.9]	86.5 ± 21.6 [85.2;79.8]
Days of sensor data	13.4 ± 2.7 [15;11]	12.7 ± 3.4 [15;9]	13.6 ± 2.9 [15;11]	13.7 ± 2.2 [14;9]
Mean CGM glucose (mg/dL)	231 ± 86 [222;339]	260 ± 97 [245;336]	255 ± 91 [241;298]	201 ± 69 [204;248]
CGM coefficient of variation	48 ± 21 [43;122]	48 ± 32 [39;122]	50 ± 22 [43;97]	47 ± 13 [45;63]
% time ≥250 mg/dL (13.9 mmol/L)	41 ± 28 [39;50]	50 ± 32 [55;49]	48 ± 28 [44;41]	32 ± 23 [34;35]
% time 181‐249 mg/dL (10.1‐13.8 mmol/L)	18 ± 9 [18;10]	16 ± 12 [14;13]	17 ± 6 [17;9]	20 ± 8 [22;8]
% time 70‐180 mg/dL (3.9‐10.0 mmol/L)	30 ± 19 [29;26]	26 ± 23 [16;25]	27 ± 19 [24;24]	31 ± 16 [31;21]
% time 55‐69 mg/dL (3.1‐3.8 mmol/L)	4 ± 5 [3;4]	4 ± 6 [2;4]	3 ± 3 [2;4]	4 ± 6 [4;5]
% time <55 mg/dL (3.0 mmol/L)	7 ± 8 [4;9]	4 ± 4 [4;7]	6 ± 9 [1;8]	6 ± 8 [6;10]

Regression analyses using CGM outcomes as the dependent variables were completed using patient demographics as the independent variables. In all cases, the data were too widely distributed to allow meaningful interpretation.

#### 3.2.1 HbA1c levels

**FIGURE 1:**
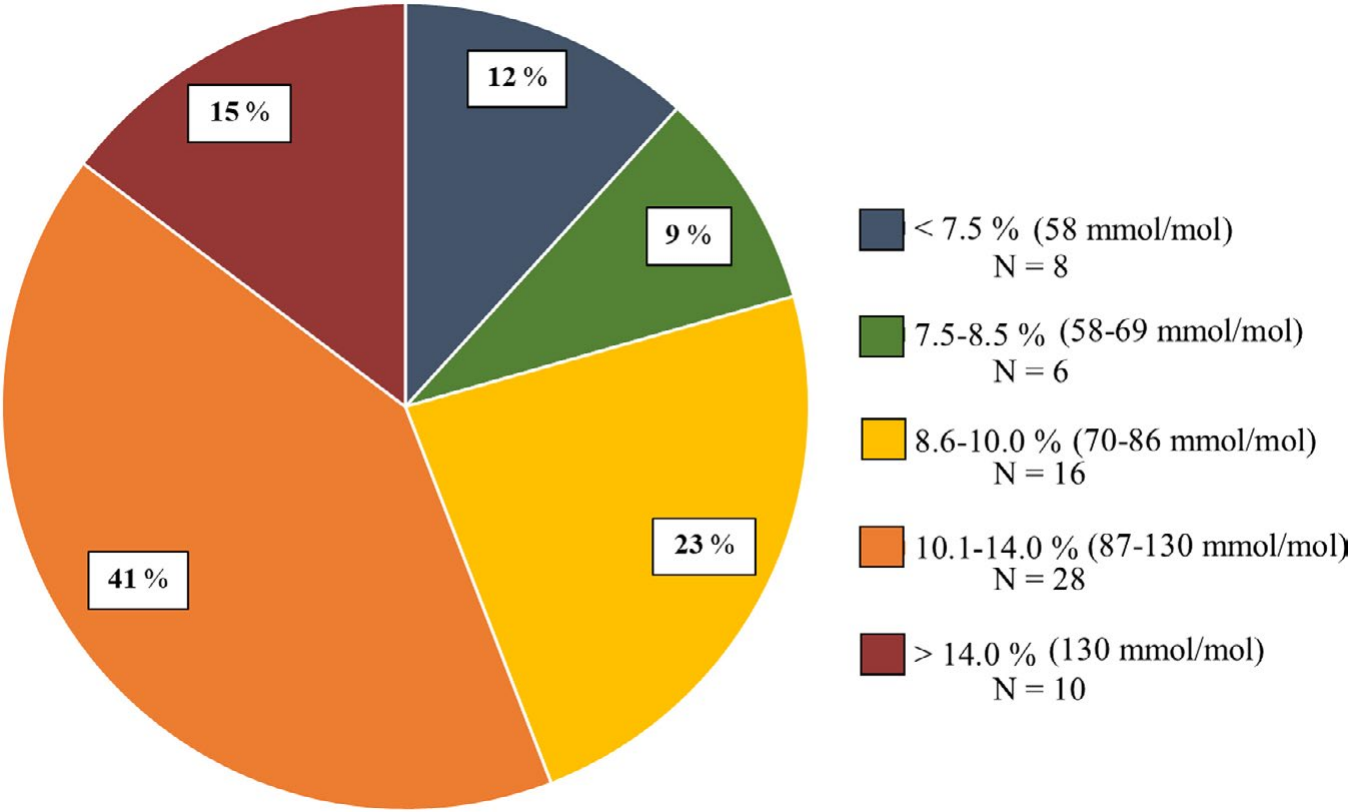
Per cent of participants in each HbA1c group

**FIGURE 2:**
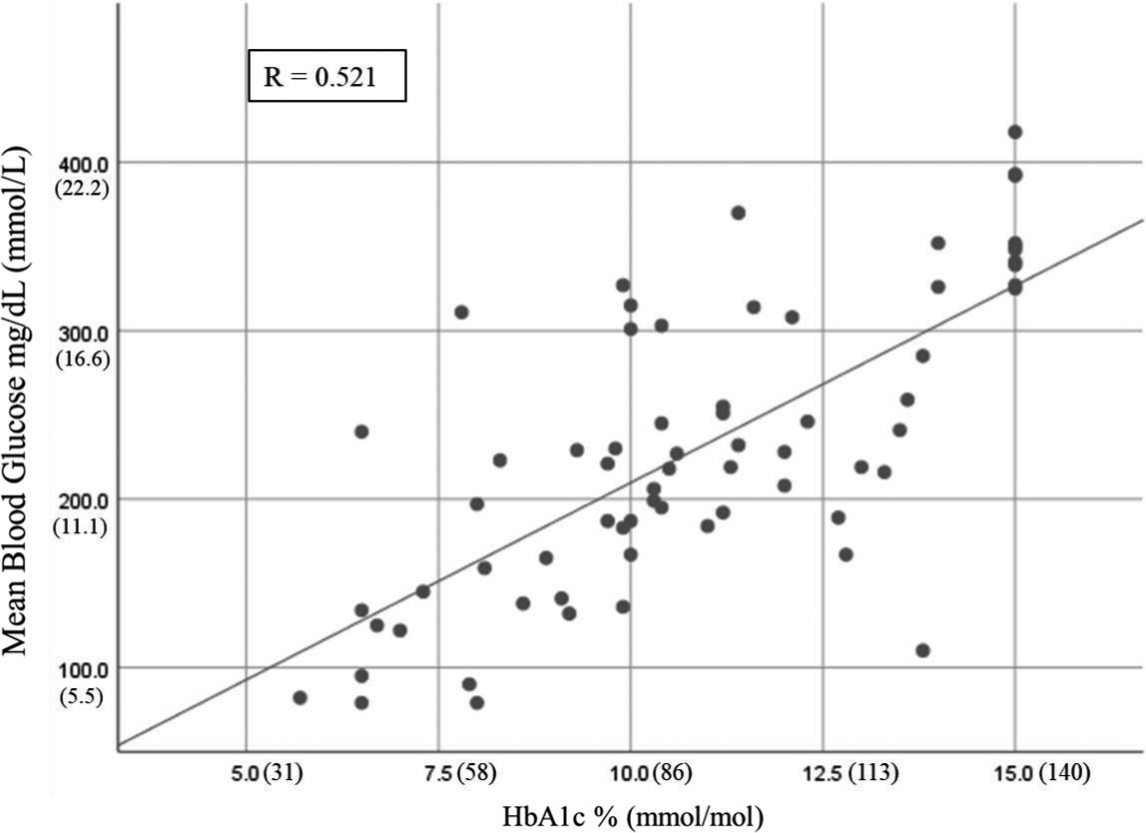
The point‐of‐care HbA1c level versus the CGM mean glucose level

Point‐of‐care HbA1c levels measured in clinic were elevated in the majority of patients (Figure 1), averaging 11.3 ± 2.7% (100 ± 30 mmol/mol) in Uganda and 9.1 ± 20% (76 ± <1 mmol/mol) in Kenya (Table 2). Levels appeared to be slightly higher in the 12‐ to 17‐year age group and lower in the 18‐ to 26‐year‐old group, but this did not achieve statistical significance. As noted in Figure 2, for each HbA1c level, there was a wide range of CGM mean glucose levels. Fifteen per cent of patients had HbA1c levels >14% (130 mmol/mol), the upper limit of measurement accuracy, and these were recorded as 15% (140 mmol/mol) for analysis purposes which may at least partially explain the glucose spread seen in Figure 2 at this highest HbA1c level.

Only 7 of 68 subjects had HbA1c values within the American Diabetes Association target range of <7.5% (58.5 mmol/mol) for children and <7.0% (53 mmol/mol) for adults.^15^ This included four children who had HbA1c levels of 5.7%‐7.0% (38.8‐53.0 mmol/mol), and three adults with HbA1c levels of 6.5%‐6.7% (47.5‐49.7 mmol/mol). The four children all had a duration of diabetes of 1 year or less and were likely in the honeymoon phase of T1D. The three adult patients, age 21‐22 years, all had a diabetes duration of >5 years and were not obese. One HbA1c level of 6.5% (47.5 mmol/mol) was likely spurious in an adult patient as her mean glucose level was 240 mg/dL (13.3 mmol/l) and she had no glucose values in the hypoglycaemic range (<55 mg/dL, 3.0 mmol/L). The other two adults with low HbA1c levels were hypoglycaemic 22%‐27% of the time.

#### 3.2.2 Blood glucose variability

Blood glucose variability, measured by CGM as the coefficient of variation (CV), has been increasingly shown to be related to morbidity. The recommended goal for CV is <36%.^16^ Mean CV was high at 48 ± 21% for this cohort. Only 13 patients had a CV <36%; unfortunately, in this population a lower CV did not usually reflect a positive pattern, as daily consistency tended to reflect consistently high numbers. Of these 13 subjects, nine had mean glucose levels of 315‐418 mg/dL (17.5‐23.2 mmol/L). Only two of the 13 had an HbA1c < 10% (85.8 mmol/mol) and seven were 14% (129.5 mmol/mol) or higher.

#### 3.2.3 Glucose time‐in‐range and time‐above‐range

**FIGURE 3:**
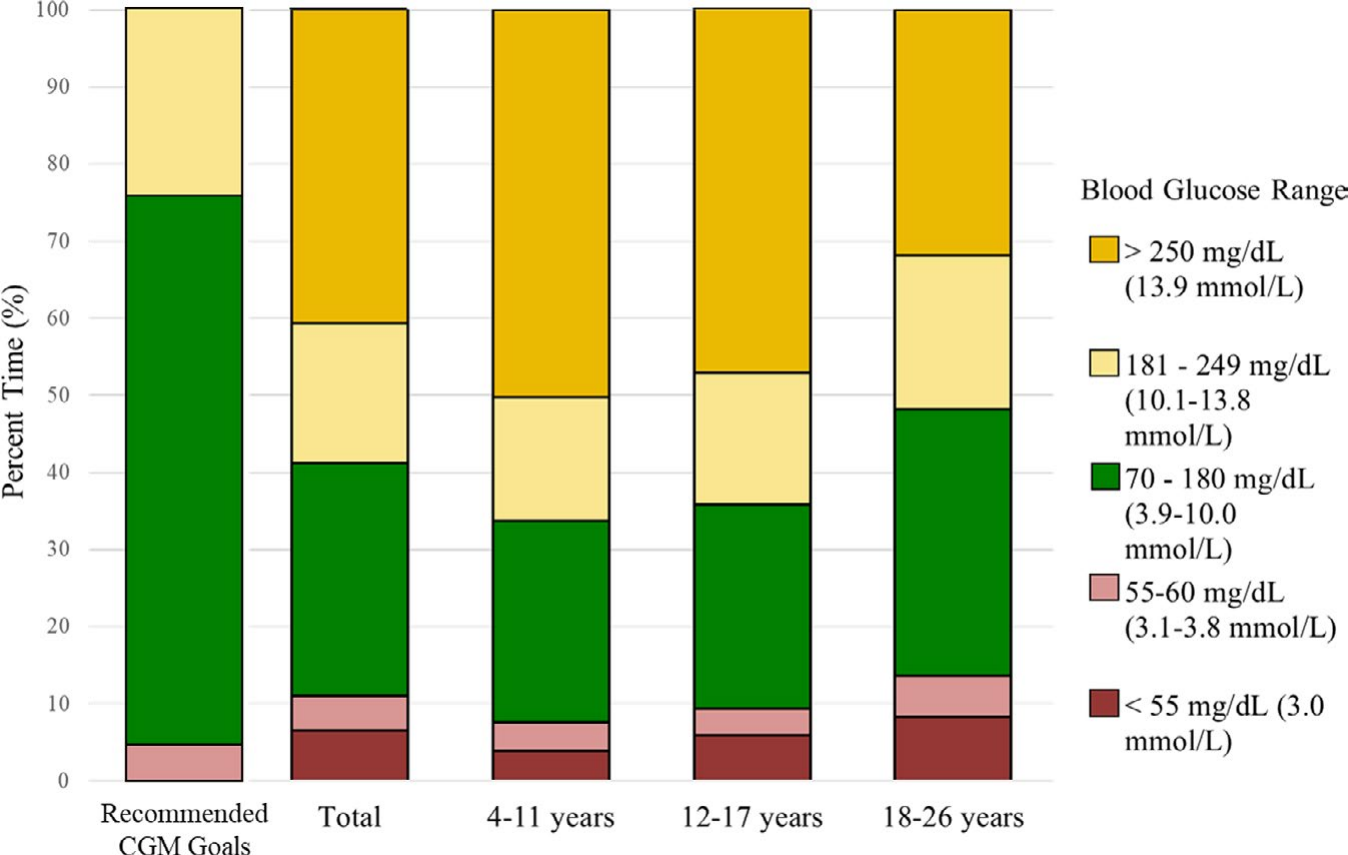
The average per cent time spent in each glucose range by CGM in 68 East African children and young adults age 4‐26 years, mean ± SD, compared to recommended CGM glucose goals^16^

**FIGURE 4:**
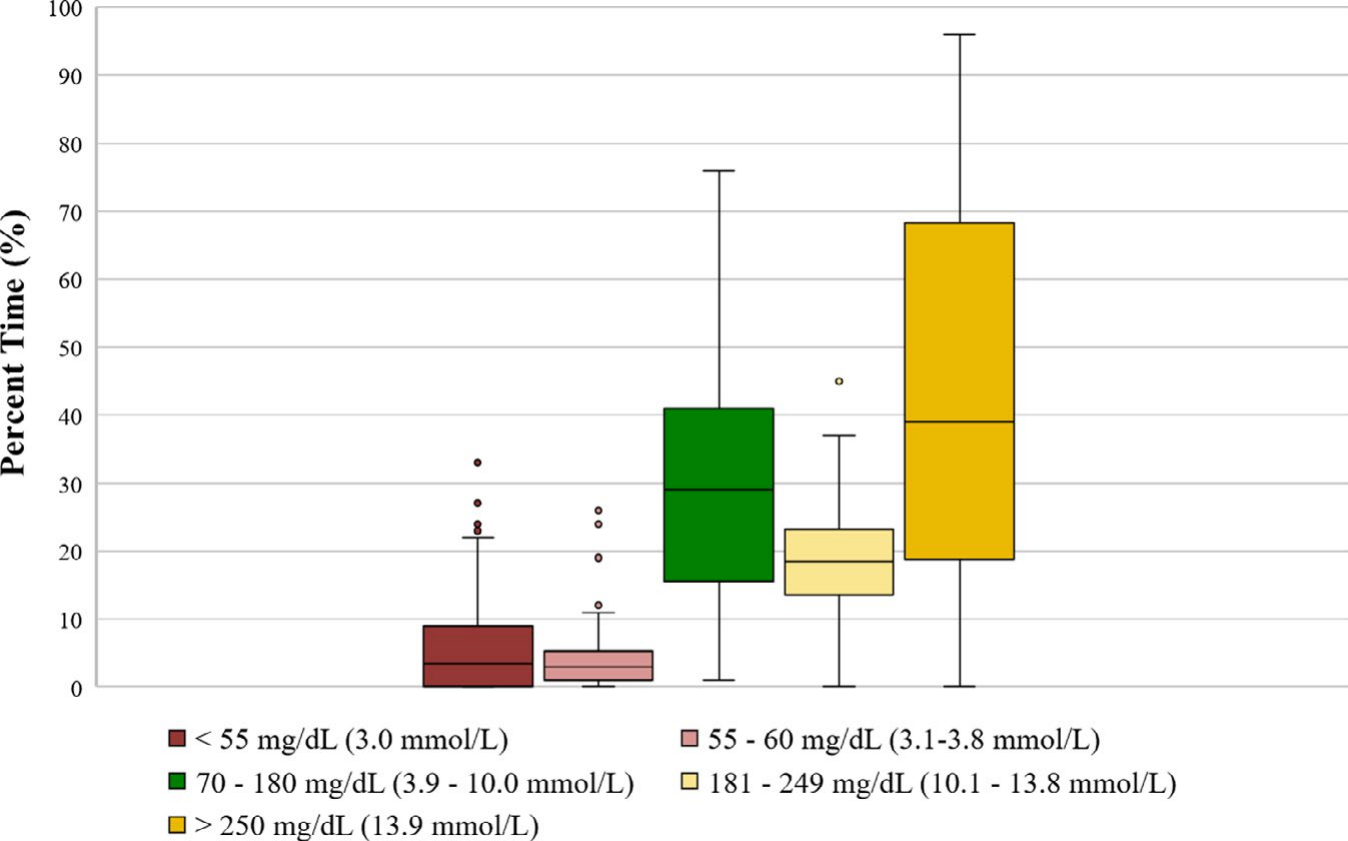
The average per cent time spent in each glucose range by CGM, expressed as median and interquartile range, N = 68

Consensus guidelines recommend that at least 70% of time be spent with glucose levels between 70 and 180 mg/dL (3.9‐10 mmol/L), considered ‘time‐in‐range’.^16^ This East African cohort demonstrated only 30 ± 19% time‐in‐range, with a nonsignificant trend towards greater time‐in‐range in the 18‐ to 26‐year‐old group (Table 2, Figure 3). Per cent time in extreme hyperglycaemia (>240 mg/dL, 13.3 mmol/L) was 41 ± 28% for the cohort as a whole. Extreme hyperglycaemia appeared to be somewhat less common in the Kenyan cohort (particularly, the young adults age 18‐26 years), but this was offset by a greater percentage of time spent in hypoglycaemia. These trends did not achieve statistical significance. Figure 4 shows the data plotted as median and interquartile range. Review of individual CGM daytime patterns revealed large postmeal spikes in almost all subjects (data not shown as it was not composite data). Notably, the morning NPH dose did not usually appear to be preventing postlunch glucose elevation.

#### 3.2.4 Hypoglycaemia

At baseline, 12 (21%) of Ugandan patients reported experiencing severe hypoglycaemia in the previous 1 year; 11 of these recalled a total of 21 episodes (1‐6 per patient), and one patient reported >15 unconscious episodes (1‐2 per month) and was forced to quit school because of this. Four Kenyan patients (33%) reported nine episodes of severe hypoglycaemia in the previous year. No patient experienced unconscious hypoglycaemia during the 2‐week study.

By CGM, over the 2‐week study period at least one hypoglycaemic event with a glucose level <55 mg/dL (3.0 mmol/L) for at least 15 minutes occurred in 81% of participants (N = 55) (Table 3). These 55 patients experienced an average of five such hypoglycaemic events per week, with a mean of almost 2 hours per day (117 ± 115 minutes) spent in the hypoglycaemic range. Clinical characteristics (age, age at diagnosis, duration of diabetes, HbA1c, mean CGM glucose, CV) did not significantly differ between subjects who did and did not experience hypoglycaemia (*P* > .05). Sensor data demonstrated that 56% of all hypoglycaemic events occurred at night (8 pm‐8 am).

**TABLE 3:** Characteristics of patients with any episode of glucose <55 mg/dL (3.0 mmol/L), mean ± SD [median; range], N = 55 (81% of cohort)

	All ages	Age 4‐11	Age 12‐17	18‐26
Uganda				
N	46	9	14	23
% time <55 mg/dL (3.0 mmol/L)	**7** ± 7	6 ± 3	7 ± 10	8 ± 7
# CGM hypoglycaemic events/wk	5 ± 4	4 ± 1	4 ± 4	6 ± 4
Average duration of each hypoglycaemic event (min)	132 ± 80	166 ± 132	134 ± 66	118 ± 59
Minutes per day <55 mg/dL (3.0 mmol/L)	117 ± 107	80 ± 41	103 ± 146	116 ± 100
Kenya				
N	9	1	2	6
% time <55 mg/dL (3.0 mmol/L)	12 ± 10	8 ± 0	12 ± 6	13 ± 12
# CGM hypoglycaemic events/wk	7 ± 5	2	7 ± 1	8 ± 6
Average duration of each hypoglycaemic event (min)	182 ± 62	293 ± 0	169 ± 76	167 ± 47
Minutes per day <55 mg/dL (3.0 mmol/L)	178 ± 137	115 ± 0	173 ± 82	190 ± 166
Combined data				
N	55	10	16	29
% time <55 mg/dL (3.0 mmol/L)	8 ± 8	6 ± 3	8 ± 10	9 ± 8
# CGM hypoglycaemic events/wk	5 ± 4	4 ± 2	4 ± 4	7 ± 5
Average duration of each hypoglycaemic event (min)	140 ± 79	178 ± 131	138 ± 65	128 ± 59
Minutes per day <55 mg/dL (3.0 mmol/L)	117 ± 115	84 ± 41	112 ± 139	131 ± 117

#### 3.2.5 Adverse events and feasibility

No major or minor adverse events related to the sensor either at insertion or during the study period were reported. Despite the fact that these are warm equatorial countries, the majority of patients did not have difficulty with sensor adhesion. They did not report significant skin irritation. The sensor was well tolerated, and many patients expressed the hope that CGM would become available to them clinically in the future.

## 4 DISCUSSION

Over the last decade, East Africa has seen major improvements in paediatric diabetes healthcare delivery, including the presence of trained paediatric endocrinology healthcare providers, education programmes and materials for providers and patients, generally sufficient quantities of human insulin and access to blood glucose monitors with about two test strips per day. Despite these positive changes, children and young adults with T1D in Uganda and Kenya struggle to maintain adequate diabetes metabolic control and HbA1c levels have remained stubbornly high. In this observational study, HbA1c levels averaged ~11% (97 mmol/mol), similar to those reported over the last 5 years in Uganda,^3^ Kenya,^5^ Tanzania^6^, ^17^ and Rwanda.^4^ Blinded CGM allowed a more granular assessment of glucose metabolic control than has previously been possible, revealing both chronic hyperglycaemia and frequent, prolonged periods of hypoglycaemia. On average, only 30% time was spent in the desired glucose target range of 70‐180 mg/dL (3.9‐10.0 mmol/L), and about 7% of time was spent <55 mg/dL (3.0 mmol/L). The average coefficient of variation (CV) of 48% is consistent with highly variable blood glucose levels, making safe insulin management extremely challenging. These patients are at high risk for serious acute and chronic complications, and it is clear that the current approach to diabetes management, although well‐intentioned, is inadequate.

Why have major improvements in diabetes healthcare delivery not translated into better diabetes metabolic control in this population? One likely factor is insufficient glucose monitoring. T1D Exchange data demonstrated that more frequent monitoring is associated with lower HbA1c levels in 1‐ to 26‐year‐olds.^18^ ISPAD 2018 Guidelines recommend fingerstick blood glucose testing 6‐10 times per day,^19^ and the American Diabetes Association recommends glucose levels be tested at least four times per day (premeal and bedtime).^15^ The current provision of about 2 test strips per day, while significantly better than in the past, is probably still not sufficient.

CGM is an alternative method of glucose monitoring that provides patients with extensive real‐time data without requiring fingerpokes or test strips. It is gaining widespread use in high‐income nations because it has been shown to decrease HbA1c and increase glucose time‐in‐range without causing hypoglycaemia.^20^, ^21^, ^22^, ^23^, ^24^ In a meta‐analysis of randomized controlled trials, the CGM‐associated reduction in HbA1c was greatest in those with the highest HbA1c at baseline. All methods of blood glucose monitoring are expensive, but until recently CGM has been more expensive than fingerpoke capillary testing. A recent report from the United Kingdom suggested that flash CGM monitoring could be cost saving for people testing blood glucose levels 6 or more times per day, especially when taking into account potential reductions in the rate of severe hypoglycaemia.^25^ While flash CGM systems are currently too expensive for common use in low‐income settings, they are significantly less costly than other CGM systems and are expected to continue to come down in price. The current study shows that CGM is feasible and well accepted by patients in a resource‐poor setting, suggesting potential future benefit both as a research tool and for clinical use.

Poor diabetes control in East Africa may be also related to problems with insulin. The average insulin dose of ~0.9 U/kg/d falls within the normal range observed for children with diabetes and does not suggest an underlying insulin resistance.^16^ Patients often do not have access to refrigeration, and they are instructed to follow East African Diabetes Study Group Guidelines which recommend insulin storage in a clean container in a cupboard at room temperature.^26^ Studies in Uganda^3^ and Kenya^5^ did not find a difference in HbA1c between patients with and without access to refrigeration, so this is not likely an issue. Rather, the problem may be with use of human insulin.

Human NPH, regular and Mixtard are the only insulins commonly available in East Africa. Pharmacologic peaks require patients to consume consistent amounts of carbohydrate at specific times, making it difficult to accommodate unanticipated changes in activity or food availability. To avoid large postprandial peaks, regular insulin should be taken at least 30 minutes before a meal, but this is often not practical. While most children in East Africa consume sufficient calories to maintain normal weight and growth, they tend to have little access to snacks between meals. They are often extremely physically active, with long walks to and from school, physical chores at home in these largely agricultural countries, and adolescent and young adult employment in physical occupations. High activity levels and limited access to between‐meal carbohydrates place them at risk for midday hypoglycaemia when the morning NPH peaks. Evening NPH peaks in the middle of the night, creating a risk for severe night‐time hypoglycaemia. Severe, life‐threatening hypoglycaemia is common in East Africa, including that reported by the current cohort (24% of patients with at least one episode in the previous year). This in turn creates a strong incentive to withhold or reduce insulin,^3^, ^27^ leading to chronic hyperglycaemia.

Would analog insulin reduce the occurrence of dangerous hypoglycaemia in East Africa and thus encourage patients and families to be more aggressive with insulin dosing? Trials of analog insulin have consistently shown a reduced risk of hypoglycaemia compared with human insulin,^28^, ^29^ and it is now standard‐of‐care for T1D diabetes treatment in high‐resource nations. In 2010, the International Insulin Foundation stated that analog insulin should be discouraged in low‐resource countries because of its expense.^30^ This statement was made a time when the commonest cause of death for a child with diabetes in Africa was lack of insulin.^31^ When children were dying because they had *no* insulin, then clearly the goal was to provide *any* insulin. However, marked improvement in insulin availability in East Africa now challenges this assumption and raises questions about the *quality* of insulin and other essential diabetes therapies, as diabetes metabolic control is still unacceptably poor.^3^, ^4^, ^5^, ^6^, ^17^

While recognizing the reality of financial limitations, it is imperative to find an appropriate balance between effective and safe treatment regimens and expense. Current practice standards arguably save costs in the short term, but the long‐term implications are bleak. With an average HbA1c ~11% (97 mmol/mol), East African children and young adults with T1D can be expected to experience acute and chronic diabetes complications which over time will result in high medical costs. There is a high cost to society as limited opportunities to obtain meaningful education and employment reduce their ability to become contributing members.^32^, ^33^ There are also high personal costs with an impact on the ability of people with T1D in these settings to marry, have children and live a satisfying life.^34^ If analog insulin was shown to reduce the risk of life‐threatening hypoglycaemia, then the ethical question would not be whether to provide this insulin in low‐income countries, but how to make it affordable. Likewise, if CGM was shown to reduce HbA1c from the catastrophically high levels currently seen, this would also have to be figured into cost‐benefit assessments. Similar cost‐benefit questions were once asked about HIV/AIDS drugs, and due to a concerted international effort, these drugs are now widely available and affordable in low‐income settings. Such decisions must be guided by data obtained in children in the specific and unique settings found in low‐income and low‐middle income nations. More research in this area is necessary.

A range of possible mean glucose concentrations for a given HbA1c level has previously been reported in T1D,^35^ especially in African American subjects.^36^ This is believed to be primarily due to individual variation in red blood cell life span.^37^ We found that HbA1c may be a particularly unreliable outcome measure in East African populations, perhaps because haemolysis from malaria is common, there is a high frequency of sickle cell trait (~13%),^38^ and there may be compromised protein nutritional status or iron deficiency, all of which can affect HbA1c values.^39^ We plan further studies to explore these relations.

There are limitations to this study. While all participants were provided notebooks and pens and asked to keep a record of insulin injections and symptoms of hypoglycaemia, only two returned with recorded information, so these data could not be included in the analysis. Smaller numbers of subjects were recruited in Kenya, and the experienced UMN team was not there to directly supervise sensor insertion that may have led to a higher rate of sensor failure. Mean glucose and A1c levels in the setting of extreme hyperglycaemia may have been underestimated, as all HbA1c values >14% (130 mmol/mol) were reported as 15% (140 mmol/mol), and the FreeStyle Libre Pro only reads glucose levels up to 500 mg/dL (28 mmol/L).

In conclusion, children with T1D in many resource‐poor regions including East Africa have seen remarkable progress in access to trained healthcare providers at dedicated diabetes clinics, engagement in diabetes education programmes, reliable availability of sufficient quantities of human insulin, and modest access to diabetes supplies. Despite these improvements, glucose metabolic control is still unacceptably poor, placing these patients at very high risk for serious acute and chronic diabetes complications. Current methods of care are not adequately serving this disadvantaged population. As new therapeutic approaches are studied, our data suggest that CGM is a feasible tool for assessing research outcomes. Furthermore, as price comes down, CGM may be a viable clinical option.

## CONFLICT OF INTEREST

Nothing to declare.

## AUTHOR CONTRIBUTIONS

AM and LMY designed the research study. All authors performed the research. LMY, EV and AM wrote the paper. All authors have edited and approved the final manuscript.

## ETHICAL APPROVAL

The study protocol received IRB approval at the University of Minnesota and each of the East African sites prior to enrolling participants. Written informed consent was obtained from all participants. Parental consent was obtained for participants aged <18 years and assent for participants age 8‐17. Consent and assent documents were provided in English and translated to the local language. Subjects were given $10 to cover travel expenses for study participation.

## Data Availability

The data that support the findings of this study are available from the corresponding author upon reasonable request.
